# Topical delivery of extracted curcumin as curcumin loaded spanlastics anti-aging gel: Optimization using experimental design and ex-vivo evaluation

**DOI:** 10.1016/j.jsps.2023.101912

**Published:** 2023-12-09

**Authors:** Rania El Hosary, Mahmoud H. Teaima, Mohamed El-Nabarawi, Yousra Yousry, Mahmoud Eltahan, Ahmed Bakr, Hussein Aboelela, Rehab Abdelmonem, Rafik M. Nassif

**Affiliations:** a Department of Pharmaceutics, Egyptian Drug Authority, Cairo, Egypt; b Department of Pharmaceutics and Industrial Pharmacy, Faculty of Pharmacy, Cairo University, Cairo, Egypt; c Department of Industrial Pharmacy, Faculty of Pharmacy, Misr University for Science and Technology, 6th October City, Egypt; d Biotechnology and Biomolecular Chemistry Program, Faculty of Science, Cairo University, Cairo, Egypt; e Faculty of Pharmacy, Misr University for Science and Technology, 6th October City, Egypt; f Department of Pharmacognosy, Faculty of Pharmacy, Misr University for Science and Technology, 6th October City, Egypt

**Keywords:** *Curcuma longa*, Curcumin, Spanlastic dispersions, Ethanol injection method, Anti-aging gel, Span® 60, Tween® 80, In-vitro release, Confocal microscopy

## Abstract

**Objective:**

This study aimed to extract and separate the organic coloring agent known as Curcumin from the rhizomes of *Curcuma longa*, and then to create Spanlastics that were loaded with curcumin using the ethanol injection technique. The optimized Spanlastic dispersions were then incorporated into a gel preparation for topical anti-aging use. The Spanlastic dispersions were analyzed for particle size, zeta potential, drug loading efficiency, and in vitro release profile. Furthermore, the rheological properties of the gel preparation were assessed, and a skin penetration study was conducted using confocal microscopy.

**Methods:**

Twelve different Curcumin-loaded Spanlastic dispersions using the ethanol injection method with Span® 60 as a surfactant and Tween® 80 as an edge activator in varying ratios. The dispersions were then subjected to various tests, such as particle size analysis, zeta potential measurement, drug entrapment efficiency assessment, and in vitro release profiling. The optimized formula was selected using Design-Expert® software version 13, then used to create a gel preparation, which utilized 2% HPMC E50 as a gelling polymer. The gel was evaluated for its rheological properties and analyzed using confocal microscopy. Additionally, Raman analysis was performed to ensure that the polymers used in the gel were compatible with the drug substance.

**Results:**

F5 formula, (that contains 10 mg Curcumin, and mixture 5 of span-tween mixtures that consist of 120 mg Span® 60 with 80 mg Tween® 80) was selected as the optimized formula with a desirability produced by Design Expert® software equal to 0.761, based on its particle size (212.8 ± 4.76), zeta potential (−29.4 ± 2.11), drug loading efficiency (99.788 ± 1.34), and in vitro release profile evaluations at Q 6hr equal to almost 100 %. Statistical significance (*P* < 0.05) was obtained using one-way ANOVA. Then F5 was used to formulate HPMC E50 gel-based preparations. The gel formula that was created and analyzed using Raman spectroscopy demonstrated no signs of incompatibility between the Curcumin and the polymers that were utilized.

The confocal spectroscopy found that the anti-aging gel preparation showed promising results in terms of skin penetration. Also, images revealed that the gel could penetrate the layers of the skin (reached a depth of about 112.5 μm), where it could potentially target and reduce the appearance of fine lines and wrinkles. The gel also appeared to be well-tolerated by the skin, with no signs of irritation or inflammation observed in the images.

**Conclusion:**

The obtained results successfully confirmed the potential of the promising (F5) formula to produce sustained release action and its ability to be incorporated into 2% HPMC E50 anti-aging gel. The confocal microscopy study suggested that the anti-aging gel had the potential to be an effective and safe topical treatment for aging skin.

## 1 Introduction

Turmeric, scientifically known as Curcuma longa L., is a highly regarded medicinal plant in traditional medicine as well as in culinary and food coloring applications. In recent times, extensive research has been conducted to explore the biological properties of turmeric (El-Saadony et al., 2023).

Turmeric contains a naturally occurring numerous phytochemicals. Among the minor constituents of turmeric, curcuminoids play a significant role and can make up as much as 10 % of dry turmeric powder (Mardani et al., 2020). It is made up of three main categories, primarily includes curcumin, dimethoxy-curcumin, and bisdemethoxycurcumin (Zhang and Kitts, 2021).

The primary curcuminoid responsible for the majority of turmeric's therapeutic properties is curcumin which has the chemical formula C_21_H_20_O_6_ and the scientific name diferuloylmethane (Roman et al., 2020). This compound is naturally hydrophobic and does not readily dissolve in water; instead, it dissolves in substances like dimethyl sulfoxide, acetone, ethanol, and oils (Ciarleglio et al., 2023).

Aging is a relatively intricate process marked by a series of events that lead to alterations in the typical functioning of an individual organism as time progresses (Troen, 2003). The development of age-related disorders is often linked to cellular dysfunction and damage caused by oxidative stress, which tends to increase as a person ages (Harman, 2002).

Over the past few years, there has been a growing interest in studying the potential anti-aging properties of curcumin. Research has shown that curcumin can help protect against these processes, promoting healthy aging by supporting cell function and preventing damage (Zia et al., 2021).

Curcumin may help protect DNA from damage caused by free radicals and other factors due to its potential role in reducing oxidative stress and inflammation (Shojaei et al., 2023).

Curcumin may help reduce protein misfolding and aggregation, which are associated with aging-related conditions by incorporating into membranes and modifying their structure, simultaneously influences the aggregation of Aβ (amyloid-beta) in an aqueous environment (Sallaberry et al., 2023).

Studies also have suggested that curcumin can support collagen production, skin health, and appearance. It contains phenolic and β-diketone functional groups that contribute to its antioxidant activity (Bērziņa and Mieriņa, 2023), which is a major contributor to skin aging. By scavenging free radicals and mitigating oxidative damage Curcumin can reduce signs of aging such as wrinkles and age spots (Jiang et al., 2020, Elkhateeb et al., 2023).

Spanlastics, nanovesicles consisting of a nonionic surfactant such as Span as bilayer forming agents and an edge activator (EA) that enhances the elasticity and flexibility of the bilayer membrane (Ansari et al., 2022). Spanlastics have recently surfaced as a potential solution for topical drug delivering, addressing the inherent challenges posed by the stratum corneum.

Due to their inherent properties, including elasticity and deformability, small nanoparticle size, drug encapsulation capabilities, and their ability to interact with the skin's lipids, Spanlastics, has shown promise in its ability to deeply penetrate the skin, effectively targeting a wide range of drugs, both hydrophilic and lipophilic, for extended periods.(Al-Mahallawi et al., 2017, Fahmy et al., 2019).

Spanlastics components, such as lipids and surfactants, can contribute to the moisturization of the skin. Proper skin hydration is essential in maintaining skin health and preventing dryness, a factor that can accelerate the appearance of fine lines and wrinkles.

A key consideration in formulating Spanlastics materials is selecting the appropriate EA that provides the necessary elasticity and deformability to navigate the tight connections of relatively impermeable membranes (Saleh et al., 2021).

Topical drug delivery offers targeted treatment, minimizing the risk of systemic absorption and related adverse effects (Marianecci et al., 2014). However, traditional gel formulations struggle to reach deep subcutaneous tissues due to limited drug spread through the skin, emphasizing the need for topically applied gels to achieve a local effect (Farghaly et al., 2017). Based on that to enhance the drug permeation and prolong skin contact, drug molecules can be encapsulated within elastic vasculature nano-carriers like Spanlastics, effectively overcoming the skin's permeability barrier.

The aim of this research was first to extract and isolate the natural dye curcumin from *Curcuma longa* rhizomes and then to develop Curcumin -loaded Spanlastics for topical delivery of Curcumin that would have improved skin penetration and controlled release, increasing the anti-aging activity of the drug.

## 2 Materials and methods

### 2.1 Materials

Curcuma longa dried rhizomes were purchased from a local herbalist from which the Curcumin was extracted. Absolute Ethanol, Span® 60, and HPMC E50 were supplied by Loba-Chemie, Mumbai, India. Tween® 80 was supplied by MP Biomedicals, USA. All the other stuff was analytical grade and used what they were given. Curcumin Standard; 500 mg, Sigma Aldrich C7727.

### 2.2 Methods

#### 2.2.1 Plant material identification

Curcuma longa rhizomes were kindly identified in the Agricultural Research Center, Cairo, Egypt by professor Dr Ibrahim El Garf.

#### 2.2.2 Extraction of Curcumin

Curcuma longa rhizome dried powder (750 g) was extracted with acetone in a Soxhlet (4 h), filtered, and evaporated (Rotavap ® Hei-VAP, Heidolph, Germany). The remained extract (65 g) was defatted with petroleum ether (3 × 1 L) and run over silica gel CC (2.5 × 60 cm, 125 g) using methylene chloride and methanol mixture as solvent system at different ratios in increasing gradient of polarity. Similar fractions were collected after comparing the TLC silica gel 60 F254 plates. Fractions were dissolved in ethanol (150 ml) and heated then methylene chloride was added (30 ml) and kept at 5 °C overnight. Yellow powder of Curcumin that precipitated were filtered, and purified on Sephadex LH-20 (110 g, 4 × 77 cm) using pure methanol (Analytical grade) and tested by spotting against standard Curcumin (500 mg, Sigma Aldrich C7727) (Revathy et al., 2011, Nurjanah and Saepudin, 2019). The extracted Curcumin is dried and weighed about 17.5 g.

#### 2.2.3 Calculation of Curcumin percentage

The percentage yield of Curcumin was calculated as follows (Schieffer, 2002).

$$Curcumin\;\%=\frac{Dry\;wt.\;of\;extracted\;Curcumin\;}{Total\;wt.\;of\;Curcuma\;longa\;rhizome\;dried\;powder\;}*100$$

$$Curcumin\;\%=\frac{17.5}{750\;}*100=2.33\%$$

#### 2.2.4 Detection of Curcumin spectrophotometrically

##### 2.2.4.1 Preparation of sample and the standard Curcumin

Standard curcumin (25 mg) weighed in a 100 ml standard measuring flask. The volume was completed to 100 ml with ethanol (0.25 mg/ml). 1 ml of this solution was withdrawn in a 100 ml standard measuring flask and completed to 100 ml (0.0025 mg/ml). The same procedures are done for sample Curcumin with the same concentration.

##### 2.2.4.2 Spectrophotometric analysis of sample and the standard Curcumin

Spectrophotometric method provides an inexpensive, rapid, specific, sensitive, precise, reliable and accurate method for the analysis of Curcumin (Kadam et al., 2018, Sravani et al., 2022).

The sample and the standard Curcumin were read by the UV spectrophotometer showing a high peak at 420 nm. Ethanol was taken as blank, while the stock standard Curcumin as a reference, and using the sample for analysis.

##### 2.2.4.3 Detection of Curcumin by TLC

Using TLC silica gel 60 F254 plates. The standard and sample Curcumin were prepared by adding 1.5 mg of each and 75 ml of ethanol into two separate vials. Using mobile phase chloroform: methanol (95:5, v/v) respectively.

#### 2.2.5 Preparation of Curcumin-loaded Spanlastics dispersions

Different types of Spanlastics containing Curcumin were formulated in the ethanol injection process as mentioned by Kakkar and Kaur (Kakkar and Kaur, 2011) using one type of span, (Span® 60) along with edge activators (Tween® 80) using two amounts of the extracted Curcumin powder 10 mg and 20 mg. Briefly, the process involved dissolving Curcumin and Span® 60 in ethanol (2 ml) and injecting it into a pre-heated, aqueous phase (10 ml) containing Tween® 80, that had already been dissolved. The organic phase to the aqueous phase ratio was fixed at 1: 5. The study examined various ratios of Span® to EA which were 200:0, 180:20, 160:40, 140:60, 120:80, and 100:100, respectively. Spanlastic Vesicles were created in a non-instructive manner and caused a slight cloudiness in the resulting hydroalcoholic solution. The solution was agitated on a magnet stirrer to guarantee that the ethanol was completely evaporated, resulting in the fabrication of Curcumin-loaded Spanlastics. Table 1 illustrates the composition of the formulae studied, which were subjected to 5 min of ELMasonic ultrasonic S 100H sonication (ELMA Corp. Germany) in order to enhance the production of superior Spanlastics dispersions.

**Table 1:** Composition of Curcumin loaded Spanlastics.

No.	Curcumin Amount(mg)	Span/Tween mixtures	Span 60 Amount (mg)	Tween 80 Amount (mg)
F1	10	Mix 1	200	0
F2	10	Mix 2	180	20
F3	10	Mix 3	160	40
F4	10	Mix 4	140	60
F5	10	Mix 5	120	80
F6	10	Mix 6	100	100
F7	20	Mix 1	200	0
F8	20	Mix 2	180	20
F9	20	Mix 3	160	40
F10	20	Mix 4	140	60
F11	20	Mix 5	120	80
F12	20	Mix 6	100	100

#### 2.2.6 Evaluation of the prepared Curcumin loaded Spanlastics

##### 2.2.6.1 Assessment of particle size, zeta potential, and PDI

The Dynamic Light Scattering technology was employed to determine the average diameter of the vesicles and the Polydispersity Index (PDI) for Spanlastic Dispersions loaded with Curcumin. The technique was based on the Zetasizer Nano ZS, Software Ver 6, 20, (Malvern instruments, Worcestershire United Kingdom). The Brownian motion of Vesicles was used to determine the z-average, a measure of the light scattering caused by Brownian motion. The measurements were triplicate at 25 °C, after the dispersion has been sufficiently diluted with the deionized water for the incident beam to achieve a scattering intensity of approximately 90° (Shailaja and Afreen, 2022).

The ELS (Electrophoretic Light Scattering) method was used to measure the zeta potentials of the systems using a laser doppler anemometer connected to the corresponding equipment. The ELS method measured the electrophoretic mobility of the vesicles under an electrical field. The measurements were made in triplicates at a temperature (25 °C) after proper dilution using deionized water.

##### 2.2.6.2 Assessment of Curcumin entrapment efficiency percentage (EE%)

To separate un-entrapped Curcumin vesicles from vesicles loaded with Curcumin, the dispersions were centrifugated at 18,000 rpm for one hour at a temperature of 4 °C. The supernatant was discarded, and the sediment was washed by redistributing the dispersions in water and centrifugating again. Because of the poor water solubility of Curcumin (0.6 µg/mL) (Górnicka et al., 2023), the washed sediment was vortex mixed with warm ethanol and allowed to sit for 15 min until completely solubilized vesicles were dissolved and Curcumin was released. The amount of entrapped Curcumin was determined spectrophotometrically at λ_max_ = 420 nm, after appropriate dilution using a Biochrom UV Libra S60 PC (Biochrom Ltd, Cambridge, UK) (Ilyas et al., 2022). The EE% was calculated according to equation (Hashim et al., 2022);

$$Entrapment\;Efficiency\;\%=\frac{Amount\;of\;entrapped\;drug}{Amount\;of\;total\;drug\;added}*100$$

##### 2.2.6.3 In vitro release studies of Curcumin from the Curcumin-loaded Spanlastic dispersions

For the in vitro release experiments, the dispersions were first loaded into a Vankel dissolution testing station (VK 7000, Vankel Industries, Inc.). The dispersions were placed into a Vankel membrane tubing called Spectra Por© (Spectra Por©, Inc., New Jersey, USA). The membrane tubing was then sealed, and the dispersions were submerged in the dissolution vessels. The dissolution medium was a 1:1 mixture of distilled water (600 ml) and ethanol (1:1) with the paddle speed set at 100 rpm. The temperature of the dissolution vessels was kept at 37 ± 0.5 °C (Niazi, 2014). 5 ml samples are taken from dispersion at specific intervals (up to 6 h) and replaced with media of equivalent volume to maintain a consistent volume. The drug release percentage is determined by a spectrophotometric analysis with λmax = 420 nm. Release studies are performed in triples. Mean drug release percentage plotted against time was obtained. A Curcumin Release Study was conducted in vitro to see if Spanlastic Systems have a retardative effect.

##### 2.2.6.4 Experimental design

The experimental data were cross-referenced using Table 2 of the Design-Expert version 13 (statistical analysis by Design-Expert, S.E Inc., Minneapolis), to investigate the influence of variables on Spanlastic dispersion particle sizes, zeta potentials, PDI, entrapment efficiencies, and in vitro release profiles after 6 h.(Albash et al., 2021).

**Table 2:** Factorial design table for Spanlastic dispersions.

**Factors**	**Factor type**	**Low Actual**	**High Actual**	**Levels**
X_1_: Drug Amount X_2_: Span-Tween mixture	CategoricCategoric	10 mgmix 1*	20 mgmix 6	2 levels6 levels
**Responses**		**Desirability constraints**		
Y_1_: Particle size Y_2_: Zeta potential (absolute values) Y_3_: Poly dispersibility index (PDI) Y_4_: Entrapment Efficiency % Y_5_: In-vitro release % (Q 6 h)		Minimize Maximize Minimize MaximizeMaximize		

* Whole experiment compositions are described in Table 1.

A factorial design (2FI) was used considering the interactions between two specific factors X_1_ (Drug Amount) and X_2_ (Span-Tween mixtures) to evaluate their combined effects on the response variables. The experiments were conducted in triplicate, and the data was presented as the mean values with standard deviation (±SD). Model terms are considered significant when the “Prob > F” values are less than 0.05.

##### 2.2.6.5 Morphologic examination via transmission electron microscopy (TEM)

After analyzing the particle size, Poly Dispersibility Index (PDI), zeta potential (ZP), entrapment (EE%), and the release profile (in vitro), the optimized Spanlastic (Curcumin)-loaded F5 dispersion, morphological analysis was performed to evaluate its structural properties (e.g. lamellarity, homogeneity of size and shape, presence of agglutinated vesicles) (Kakkar and Pal Kaur, 2013). In a brief manner, a microgram (0.1 ml) of Spanlastic dispersion was applied to a 300-mesh carbon coated copper grid and left to settle for a period of 3 to 5 min. The excess fluid was then removed with filter paper. The sample was allowed to air dry for 10 min at room temperature before being subjected to TEM under a JEM 1230 (Tokyo, Japan) at a voltage of 80 kV.

##### 2.2.6.6 Gel preparation using the optimized formula (F5)

The design expert software version 13 was utilized to determine the optimal Spanlastics formula which was then applied to the gel preparation process. The gel was initially prepared by adding the desired amount of 2 % HPMC E50 (0.2 g) to a sufficient portion of distilled water (10 ml) and stirring at an internal temperature of 80 °C (Uthaiwat et al., 2021). Subsequently, adequate amount of the optimized Spanlastic-loaded Curcumin dispersions was added to the prepared gel base then the volume was adjusted to 100 ml by using distilled water so that the final formulation contained 10 mg of Curcumin per 1 g of gel base which is the therapeutic dose of Curcumin for topical applications (Panda, 2018). The gels were then stored overnight in the refrigerator for further evaluation.

#### 2.2.7 In-vitro evaluation of the prepared gel

##### 2.2.7.1 Visual inspection

The gel was presented against a black-and-white background to allow visual inspection of the appearance and properties of the gel, such as color, precipitation, and homogeneity.

##### 2.2.7.2 The pH measurement

A digital pH meter was used to measure the pH of the gel. 1 g of gel is reconstituted with 9 ml of distilled water. The dilution is done with a magnetic stirrer and, if necessary, heated over water bath. The pH of the solution is measured after cooling to room temperature. The gel is stored for about 2 h.

##### 2.2.7.3 Drug content determination for the prepared gel

To find the average drug content of the gel, one gram of gel was dissolved in 100 ml ethanol. Aliquots were withdrawn and filtered before being diluted. The absorbance was measured in triplicates at a λ_max_ of 420 nm and the mean drug content was calculated.

##### 2.2.7.4 Rheological study of the prepared gel

Measurement of viscosity was performed using a viscometer cone and plate model III. The pre-processed gel was placed into a viscometer cup. Spindle 52 was employed at a speed of 25 °C (±1 °C) and (25–250 °C).

##### 2.2.7.5 Confocal laser scanning microscopy study (CLSM)

The full body skin of six Wistar rats weighing between (200 – 250) g was excised and used after euthanization through cervical dislocation. If there was subcutaneous tissue, it was eliminated, and the dermal side was cleansed with isopropyl alcohol to eliminate any attached fat. Subsequently, the skin underwent a saline wash and visual inspection to confirm the absence of any incisions (Farghaly et al., 2017). At the end of the study, the remains of rats had been frozen and transferred to be incinerated at Faculty of Veterinary Medicine, Cairo University. The protocol for this study has been reviewed and approved by the research Ethics Committee of the Faculty of Pharmacy, Cairo University, (PI 1405) Egypt. This protocol is in accordance with the Animal Care and Use Guidelines published by the Institute of Laboratory Animal Research (Washington D.C., United States).

CLSM is an imaging method in which a spatial pinhole is used to remove the out-of-focus light during image creation. CLSM allows for accurate and well-defined depth of focus. To evaluate the selected Spanlastic-loaded gel formula with Curcumin against Curcumin suspension as a control, 0.05 % of Rhodamine B dye were loaded and applied to the rats' skin that has been cut off. The rats' skin was cross-sectioned mechanically using a specially designed cross-section device (van Kuijk-Meuwissen et al., 1998), and mounted homogeneously onto the Franz diffusion cell for 6 h. The skin is then scraped, and a slide is generated. The slide after that is tested with a Confocal Microscope (Leica TCS Spectral CLSM 710, UK) using argon laser beam (excitation wavelength: 488 nm, emission wavelength: 590 nm) (Ansari et al., 2022).

##### 2.2.7.6 Compatibility studies of optimized Curcumin-loaded Spanlastic dispersion (F5) with the formulated additives

Raman spectrometer was used to evaluate the potential interactions of Curcumin with other compounds in the optimized Curcumin loaded Spanlastic dispersion (Allakhverdiev et al., 2022). The Raman spectra of the optimized Curcumin-loaded Spanlastic dispersion (F5) were obtained using a Horiba lab RAM HR evolution visible single spectrometer in Edison, NJ, USA. Measurements were made at room temperature with a 532 nm HE-CD edge laser line with 1800 grating at 450–850 nm and a ND filter of 0.01 %. Acquisition time was 20 s, no delay time, and no spike filter. Measurements were done in the range of 100–3199 cm^−1^, and any changes, spikes, or gradients were noted.

While, distinct peaks of Curcumin, such as 959 cm^−1^ for C = O, 1599 cm^−1^ for C = C aromatic, 1413 and 1429 cm^−1^ for Phenol C-O, and 1248 cm^−1^ for Enol C-O were reported by *van Nang et al* (Van Nong et al., 2016).

## 3 Results

### 3.1 TLC identification of Curcumin

**Fig. 1:**
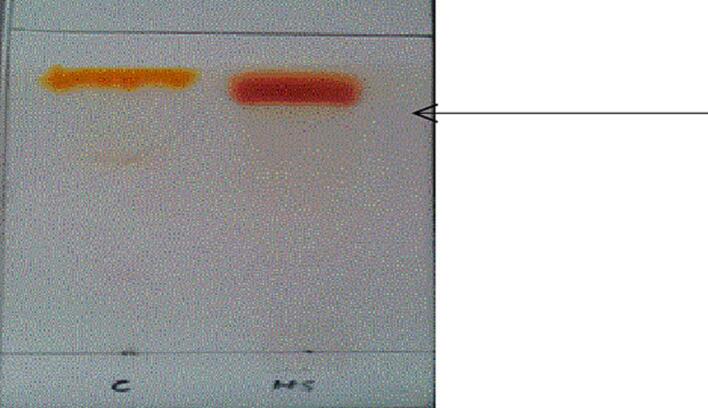
TLC result of Curcumin sample extracted from Curcuma longa. L and standard curcumin.

Using chloroform and methanol in a ratio of 95:5 respectively as mobile phase. The sample was run on TLC as shown in Fig. 1 against standard curcumin, we got single spot of RF value at 0.59. According to the RF value of standard curcumin we analyzed presence of curcumin in the sample.

### 3.2 Spectrophotometric analysis

**Fig. 2:**
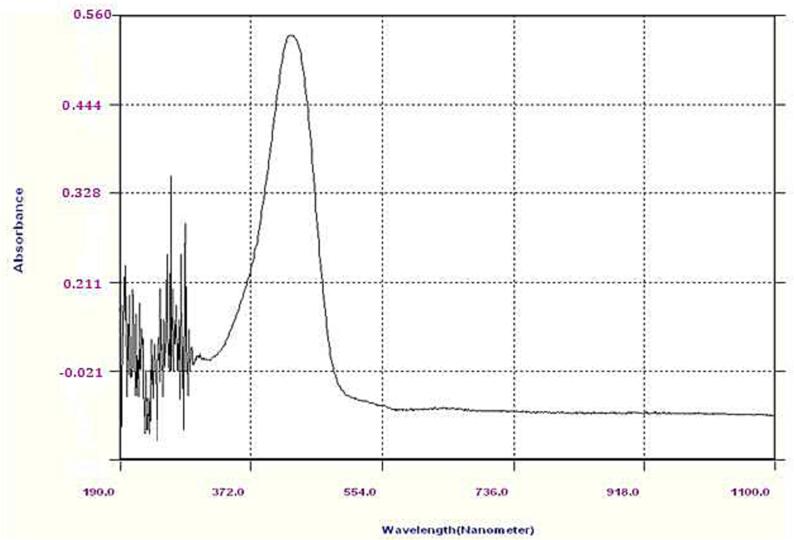
UV absorption spectra of curcumin standard.

**Fig. 3:**
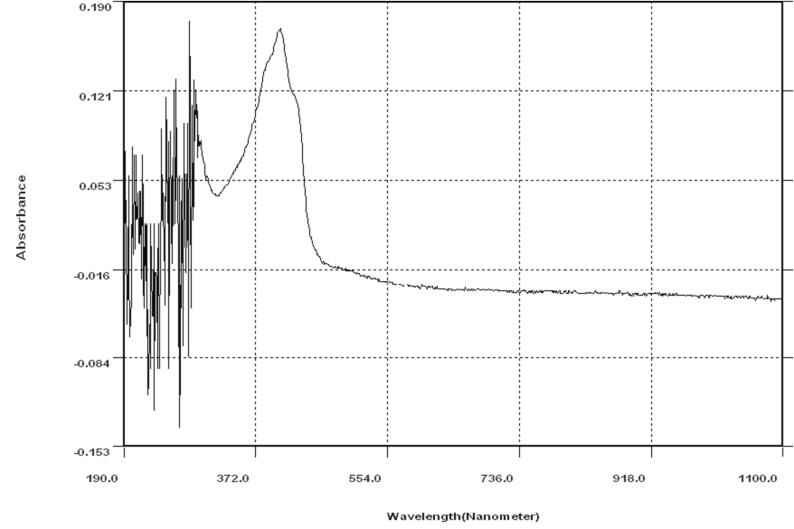
UV absorption spectra of curcumin extracted from *Curcuma longa.* L.

The chromatograph for standard solution is obtained as shown in Fig. 2, Fig. 3.

Fig. 2 shows the maximum absorbance of the standard curcumin at the wavelength of 420 nm and absorbance of 0.545. Similarly, Fig. 3 shows curcumin sample extracted from Curcuma longa. L with maximum absorbance at the wavelength of 420 nm and absorbance of 0.174.

### 3.3 Evaluation of the prepared Curcumin loaded Spanlastics

#### 3.3.1 Determination of particle size, zeta potential, and polydispersity index

**Fig. 4:**
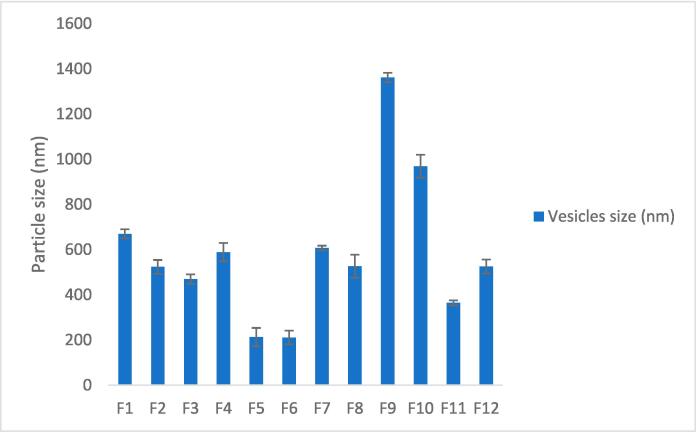
Particle size of Curcumin-loaded Spanlastics.

Table 3 displays the mean values for particle size, zeta potential, and PDI of the developed Spanlastic formulae, and all parameters had a significant effect (P < 0.05). The results of Fig. 4 demonstrate that the average particle size for the first six dispersions (F1 through F6) varied from 669.7 nm (F1) to 210.9 nm (F6) respectively.

**Table 3:** The particle size, zeta potential values, PDIs, and EE% of Curcumin-loaded Spanlastic formulae.

No.	Vesicles size(nm)	Zeta potential(mV)	Poly Dispersibility Index	Entrapment Efficiency %
F1	669.7 ± 2.34	−46.1 ± 4.23	0.845 ± 0.112	99.58 ± 1.67
F2	523.4 ± 3.89	−33 ± 2.46	0.651 ± 0.078	98.942 ± 1.32
F3	469.5 ± 2.56	−32.8 ± 3.12	0.616 ± 0.056	98.8 ± 1.06
F4	588.8 ± 4.21	−35.5 ± 3.67	0.712 ± 0.099	98.77 ± 1.89
F5	212.8 ± 4.76	−29.4 ± 2.11	0.379 ± 0.043	99.788 ± 1.34
F6	210.9 ± 3.45	−32.3 ± 2.34	0.409 ± 0.064	99.106 ± 1.56
F7	607 ± 1.23	−43.4 ± 3.21	0.611 ± 0.032	98.98 ± 1.23
F8	526.6 ± 5.98	−36.9 ± 2.98	0.71 ± 0.087	98.42 ± 1.76
F9	1361 ± 2.67	−30.7 ± 2.67	1 ± 0.076	98.27 ± 1.45
F10	969.3 ± 5.32	−23.7 ± 4.56	0.987 ± 0.123	98.04 ± 1.89
F11	364.2 ± 1.87	−31.5 ± 2.34	0.561 ± 0.045	98.06 ± 1.23
F12	525 ± 3.62	−34 ± 3.45	0.679 ± 0.053	97.77 ± 1.56

All data are represented as (mean ± S.D., n = 3).

**Fig. 5:**
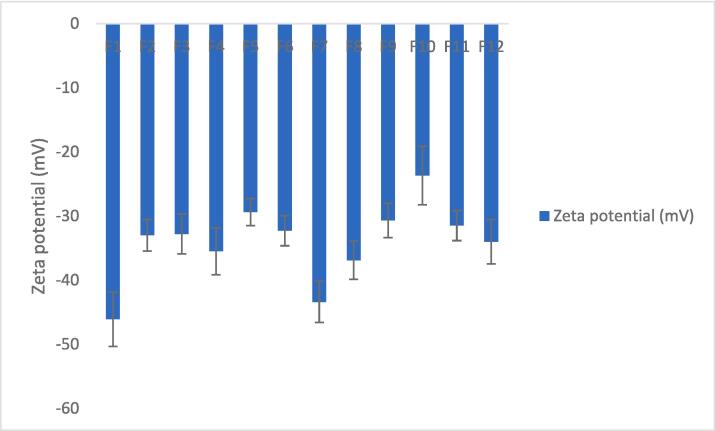
Zeta potential values of Curcumin–loaded Spanlastics.

Spanlastic dispersions all exhibit negative Zeta potentials, with F1 having a negative Zeta potential of −46.1 mV and F10 having a negative zeta potential of −23.7 mV.

#### 3.3.2 Estimation of Curcumin EE %

The EE percentages of Curcumin-loaded Spanlastic dispersions were significantly varied (F value = 428.86, P < 0.05) ranging from 99.788 % for F5 to 97.77 % for F12 as shown in Table 3 this may be due to the lipophilic nature of Curcumin that synergized the activity of Span 60® which promote the formation of stable vesicles in a highly effective entrapment (EE%) due to its lipophilic nature and saturated alkyl chains as reported in the article entitled “Spanlastic nanovesicles for enhanced ocular delivery of vanillic acid: design, in vitro characterization, and in vivo anti-inflammatory evaluation” (Elsaied et al., 2022).

#### 3.3.3 In vitro release studies of Curcumin from the Curcumin-loaded Spanlastic dispersions

**Fig. 6:**
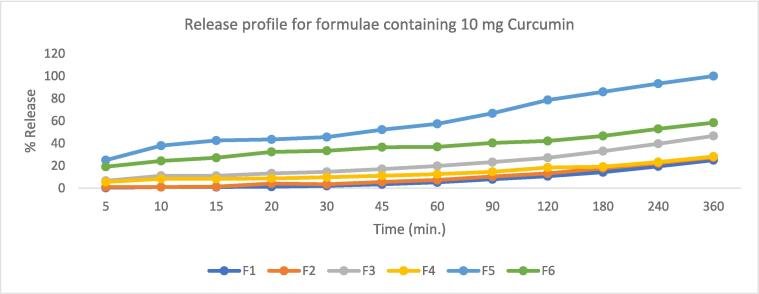
Curcumin release profile from formulae containing 10 mg Curcumin.

**Fig. 7:**
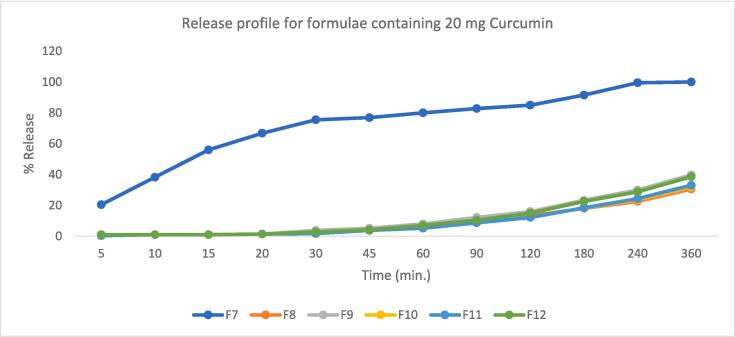
Curcumin release profile from formulae containing 20 mg Curcumin.

The in vitro drug release studies of Curcumin-loaded Spanlastic dispersions are represented graphically in Fig. 6, Fig. 7. The release % of Curcumin-loaded Spanlastic dispersions varied significantly (F value = 4178.85, P < 0.05) at Q 6 h (after 6 h) from 25 % for F1 to 100 % for both F5 and F7. Span 60® to EA ratio significantly affected Q6h. Also results showed that Spanlastics prepared using a 9:1 ratio released significantly lower amounts of the drug after 6 h. in our case both ratios10:0, and 9:1 show the same results, and the other ratios proved to be non-significantly different from one another (Fahmy et al., 2019). While in case of formulae containing 20 mg Curcumin the release profile was markedly lower than that containing 10 mg only, this may be due to the lipophilic nature of Curcumin that binds tightly with the lipophilic Span as discussed before yielding lower amount of the released drug.

#### 3.3.4 Experimental design

The predicted and adjusted R^2^ values were statistically accepted as shown in Table 4, so there were no issues with the data or model. (Albash et al., 2021).

**Table 4:** Output data of the Full factorial design analysis of prepared Spanlastic dispersions.

Response	Y1: Particle size	Y2: Zeta potential	Y3: PDI	Y4: EE %	Y5: In-vitro release % (Q 6 h)
Minimum Maximum R-squared Adj. R^2^ Pre. R^2^Adequate precision	210.80 1362 1.0000 1.0000 1.00002269.210	−23.60 −46.20 0.9947 0.9899 0.978853.546	0.38 1.10 0.9761 0.9543 0.904621.395	97.76 99.79 0.9975 0.9951 0.989967.257	24 100 0.9997 0.9995 0.9990182.327

**Fig. 8:**
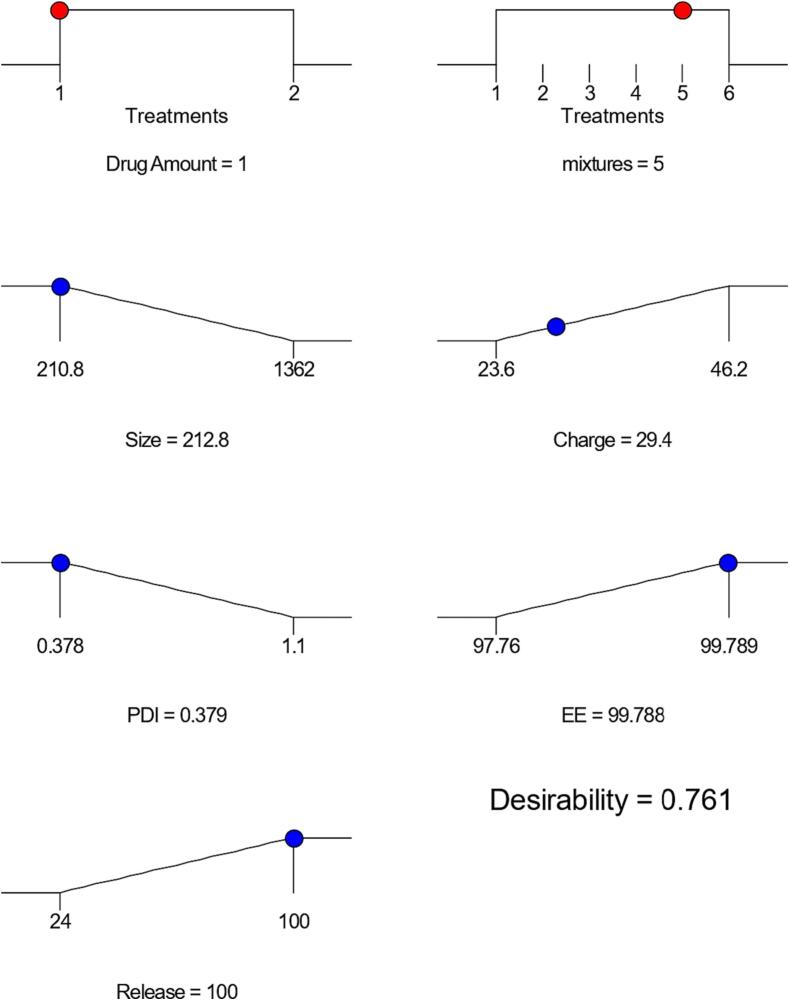
Ramps of desirability for the optimized Formulation (F5).

**Fig. 9:**
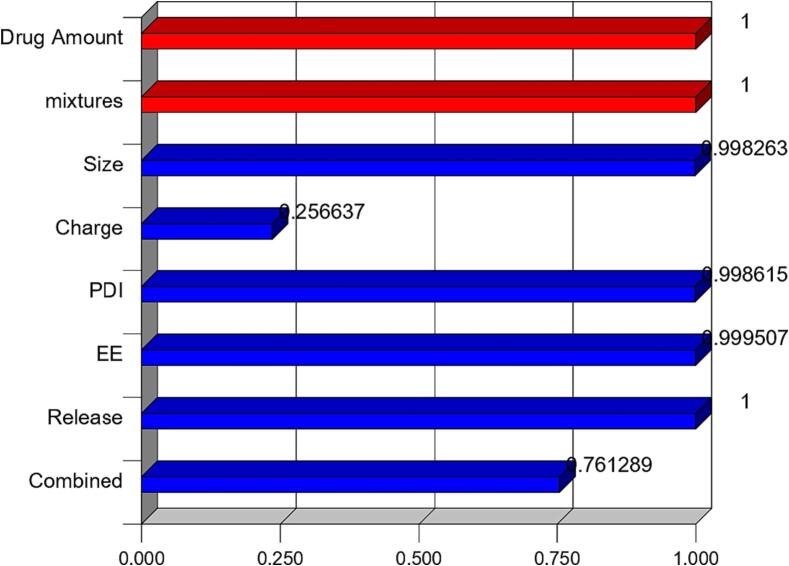
Bar graph of the desirability for the optimized Formulation (F5).

Table 5 of the Desirability report prepared by Design Expert (version 13) shows that the optimal formulae chosen based on different parameter(s) (particulate size, Zeta potential, Poly dispersibility Index, Entrapment Efficiency Percentage, In Vitro Release Profile at Q6h) was F5 (Desirability = 0,761) as shown in Fig. 8 and Fig. 9.

**Table 5:** Solutions table for 12 combinations of the optimization process.

Number	X1: Drug Amount	X2: Span-Tween mixture	Y1: Particle size	Y2: Zeta potential	Y3: PDI	Y4: EE %	Release %(Q 6 h)	Desirability	
1	10 mg	mix 5	212.8	−29.4	0.379	99.788	100	0.761289206	**Selected**
2	20 mg	mix 1	607	−43.4	0.611	98.98	100	0.747894731	
3	10 mg	mix 6	210.9	−32.3	0.409	99.106	58.46	0.644044278	
4	10 mg	mix 3	469.5	−32.8	0.616	98.8	46.6	0.503150869	
5	20 mg	mix 2	526.6	−36.9	0.71	98.42	30.62	0.36564877	
6	10 mg	mix 4	588.85	−35.5	0.712	98.77	28.18	0.34934104	
7	10 mg	mix 2	523.4	−33	0.651	98.942	27.5	0.347331765	
8	20 mg	mix 5	364.2	−31.5	0.561	98.06	33.06	0.331225184	
9	10 mg	mix 1	669.7	−46.1	0.845	99.58	25	0.301605511	
10	20 mg	mix 6	525	−34	0.679	97.77	38.62	0.179233523	
11	20 mg	mix 4	969.3	−23.7	0.987	98.04	32	0.080741918	
12	20 mg	mix 3	1361	−30.7	1	98.27	39.7	0.072204992

#### 3.3.5 In-vitro evaluation of the prepared gel

##### 3.3.5.1 Visual inspection

The prepared gel was homogenous, translucent, and didn’t precipitate.

##### 3.3.5.2 The pH measurement

The gel was found to have a pH value of 6.8 ± 0.14, which was considered suitable and non-irritating to the skin surface, as reported by *Al-Nima et. al* (Al-Nima et al., 2019).

##### 3.3.5.3 Drug content determination for the prepared gel

Drug content percentage value ranged found to be 97.3 ± 1.21 %.

##### 3.3.5.4 Rheological study of the prepared gel

**Fig. 10:**
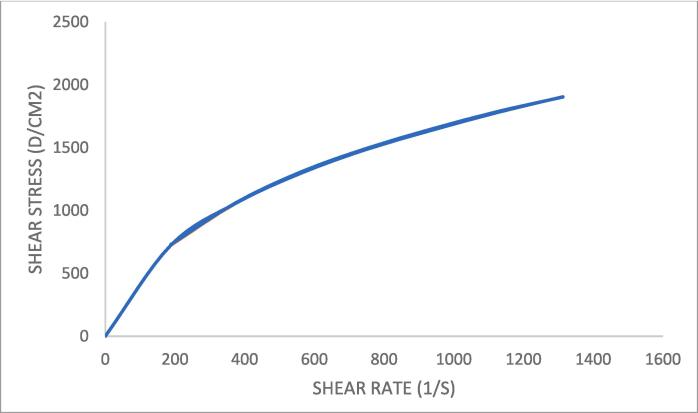
A rheogram for the prepared gel.

Fig. 10 shows thixotropic behavior of the prepared formula with the shear-thinning pseudoplastic flow. The viscosity decreased as the shear rate increased.

#### 3.3.6 Morphologic examination via TEM

**Fig. 11:**
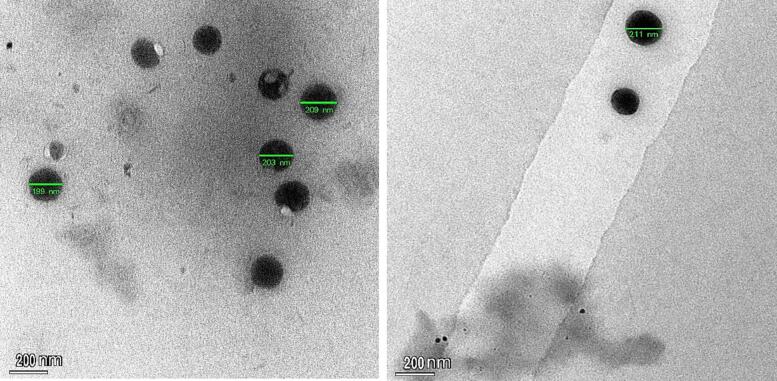
TEM image of F5 (Curcumin-loaded Spanlastics).

TEM was used to investigate the morphology of the optimized Spanlastics nano-formulation. As shown in Fig. 11, the hypothesized nano formulation consisted of non-aggregated, dispersed, and spherical particles with a flat surface (Badria and Mazyed, 2020). On the other hand, the photomicrograph showed that the Spanlastics are in the nanometer range and that all the particles fall within the size range as resulted by zeta sizer, which is less than 213 nm. The size of the particles observed under TEM is in line with the mean size (212.8 nm) as reported using zeta sizer.

#### 3.3.7 Confocal laser scanning microscopy study

**Fig. 12:**
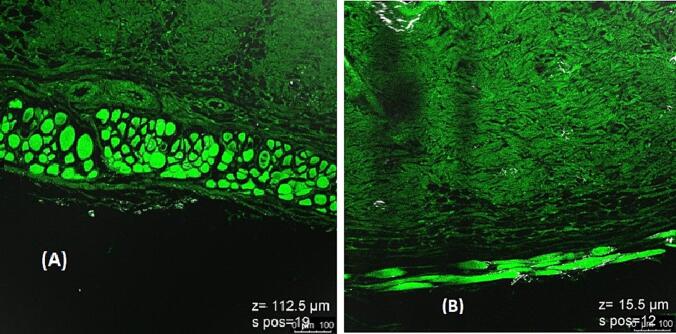
CLSM image obtained for (A) the optimized formula (F5) and (B) for the Curcumin-suspension (control).

As shown in Fig. 12, the Curcumin-loaded Spanlastics (A) were observed to penetrate deeper through the layers of skin compared to the Curcumin-suspension (control) (B) in a CLSM-aided skin penetration experiment (Kapoor et al., 2019). The Spanlastics after 6 h reached a depth of about 112.5 μm, while the Curcumin-suspension (control) was confined to a depth of only 15.5 μm.

#### 3.3.8 Compatibility studies of optimized Curcumin-loaded Spanlastic dispersion (F5) with the formulated additives

**Fig. 13:**
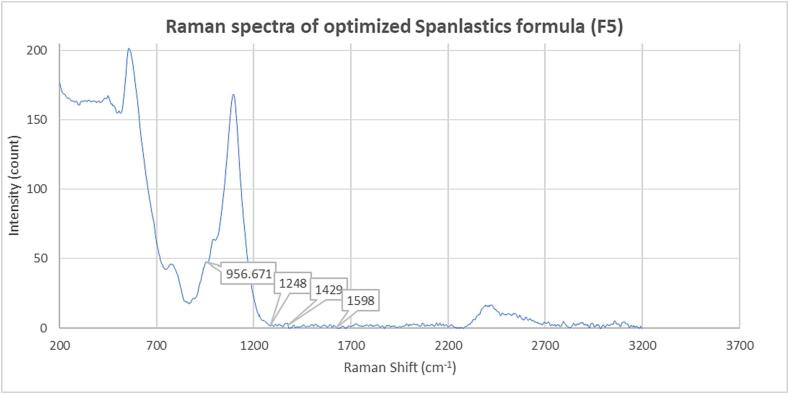
Raman spectra of optimized Spanlastics formula (F5).

Raman spectroscopy was performed on the optimized Curcumin-loaded Spanlastics (F5), as shown in Fig. 13, and no significant changes, peak shifts, or broadening were observed in the distinct peaks of Curcumin, or the excipients. This indicates that there was no chemical interaction between the excipients and Curcumin.

## 4 Discussion

In relation to particle size, the incorporation of edge activator (EA) Tween® 80 had a positive impact on diminishing aggregation tendencies, resulting in vesicles with smaller diameters compared to those without the EA (F1 or F7) dispersions (Rosen and Kunjappu, 2012).

The concentration of the EA exhibited an inverse correlation with the mean particle size, with the smallest vesicles produced at a 1:1 ratio of Span® to EA (F6). This may be attributed to higher concentrations of edge activators possessing greater emulsification power (Elmowafy et al., 2019).

However, at lower concentrations, they may prove insufficient to coat the entire vesicle surface. Consequently, vesicles can accumulate until the surface area is adequately reduced for the surfactant to cover it entirely, resulting in a stable dispersion containing relatively large vesicles (Almuqbil et al., 2022).

Similar findings were reported by (Dora et al., 2010), where a higher amount of EA decreased surface tension, facilitating particle separation and the formation of smaller nano-glibenclamide.

Additionally, an increase in Span® concentrations led to an augmentation in vesicle size. This was attributed to the introduction of larger alkyl groups from Span® into the hydrophilic domain of vesicles, thereby diminishing the interaction between the polar heads of edge activators (Elsherif et al., 2017).

In the second set of six dispersions (F7 to F12), the observed variation in the effects of Span and Tween mixtures is likely due to interference caused by an increased drug quantity. This increase necessitated a higher amount of surfactant to cover the drug adequately, resulting in a stable Spanlastic dispersion with relatively larger particle-sized vesicles. This aligns with findings reported by (Quion and Jones, 1994).

Smaller particles, with their increased surface area, generally exhibit enhanced skin penetration capabilities by better interacting with the skin barrier. They are more likely to permeate the outer layers and reach deeper tissues. In contrast, larger particles may encounter challenges in crossing the skin barrier (Fahmy et al., 2019).

As shown in Table 3 and Fig. 5, the negative zeta potentials decrease as the EA concentration increases. This is due to the accumulation of hydrophilic edge activation on the surface of vesicular bilayers which shields the negative surface charges depending on the concentration (Ibrahim and Abd-Allah, 2022).

At lower ratios of Span® to EA (6:4, 1:1), PDI values were found to be lower, indicating the creation of small, evenly distributed vesicles. This was observed in samples F5, F6, and F11 (Elsaied et al., 2022).

The negative zeta potential of the produced Spanlastics can contribute to repulsion between particles, enhancing stability and potentially influencing interactions with skin surfaces (Elhabak et al., 2021).

In experimental design adequate precision ensures that the model is able to navigate the design area when the value of signal: noise ratio exceeds four, this was confirmed in all results (Abdolkarimi and Mosavi, 2020). While, the R^2^ is an indicator of the design's ability to anticipate the values of various responses. (Gürel et al., 2020).

Rheological study of the prepared gel resulting a pseudoplastic flow, this may be due to the gradual rupture of the formula's internal structure under increasing shear and subsequent reconstruction through Brownian movement. (El-sherif et al., 2018, Panraksa et al., 2020).

In CLSM study, the optimized Spanlastics formulation was able to easily penetrate this barrier and reach the deeper layers of the skin. In addition, the elastic nature of the vesicles may have contributed to their ability to penetrate deeper into the skin.

## 5 Conclusion

Curcumin was successfully extracted from Curcuma longa rhizomes using acetone in Soxhlet. Curcumin-loaded Spanlastic dispersions were prepared using Span® 60 and Tween® 80 via the ethanol injection method. Formula (F5) with 10 mg Curcumin and a 6:4 ratio of Span to Tween was identified as the optimal Spanlastic formula, demonstrating superior particle size, zeta potential, drug loading efficiency, and in vitro release. This formula was used to create an anti-aging gel with 2 % HPMC E50. Raman spectroscopy confirmed compatibility between Curcumin and the polymers. Confocal spectroscopy showed promising skin penetration and deeper layers' targeting. The gel was stable and well-tolerated, with no signs of irritation or inflammation in further evaluations.

## Declaration of competing interest

The authors declare that they have no known competing financial interests or personal relationships that could have appeared to influence the work reported in this paper.
